# Recapitulation of Ageism in Artificial Intelligence–Generated Images: Longitudinal Comparative Study

**DOI:** 10.2196/68428

**Published:** 2025-08-13

**Authors:** Lindsey Martens, Nicole Virgin, Phillip Hoffarth, Jade Goodwill, Nicole Derenne, Richard Van Eck, Marilyn Klug, Gunjan Dhawan Manocha, Donald Jurivich

**Affiliations:** 1 Department of Geriatrics, School of Medicine and Health Sciences, University of North Dakota, 1301 N Columbia Rd, Grand Forks, ND, 58202, United States, 1 7017774455; 2 Department of Arts and Design, University of North Dakota, Grand Forks, ND, United States; 3 Department of Population Health, School of Medicine and Health Sciences, University of North Dakota, Grand Forks, ND, United States

**Keywords:** artificial intelligence, aging, ageism, digital ageism, geriatrics

## Abstract

**Background:**

Positive images of aging in traditional media promote better health outcomes in older adults, including increased life expectancy. Images produced by generative artifical intelligence (AI) technologies may reflect and amplify societal age-related biases, a phenomenon known as digital ageism. This study addresses a gap in research on the perpetuation of digital ageism in AI-generated images over time.

**Objective:**

This study examined how visual characteristics of digital ageism in AI-generated representations of older adults changed over time. It aims to provide insight into the interplay between technology advancements, societal attitudes toward aging, and the well-being of older adults interacting with digital media.

**Methods:**

This longitudinal study compared 164 images generated by Open AI’s DALL-E 2 at 2 time points, 1 year apart (2022 and 2023). Identical text prompts from the geriatric lexicon (eg, *frail older adult*, *dementia*) were used at both time points. Authors evaluated the images generated for demographic characteristics (perceived gender, race, and socioeconomic status), and primary emotion characteristics, then compared the frequency of these characteristics between years and evaluation characteristics using a type III 2-way ANOVA.

**Results:**

Representations of White-racialized older adults were 5-fold higher than those of other races in both years. The mean number of representations of Asian-racialized individuals increased from 20 to 31 (*P*=.004), and the mean number of other racialized representations also increased, from 6 to 14 (*P*=.007). Representations of people with a middle-class socioeconomic status were significantly more frequent than other statuses in 2022 and 2023 with no changes in socioeconomic status from one year to the next. Prompts were largely neutral for expression terms, while image analyses for expressions did not show significant differences in positive, neutral, or negative emotions between 2022 and 2023. Prompts used for image generation had more male-oriented terms than expected, and male representation was higher then female representation in the images, with no difference in sex representation between the 2 time points.

**Conclusions:**

Despite a social emphasis on positive views on aging, AI text-to-image generators persistently generated images with characteristics of digital ageism. Images predominantly featured White-racialized individuals at both time points, with no improvement in emotional representation despite using neutral text prompts. These findings highlight the persistence of ageist visual characteristics in AI-generated images over time. A limitation of this study is that it focused only on AI image generation and did not analyze other AI-generated content that may express digital ageism.

## Introduction

Ageism was defined in 1969 to recognize biases and discrimination based on chronological age [1]. Today, the World Health Organization (WHO) defines ageism as prejudice, stereotypes, and discrimination directed toward individuals based on age [2]. More than 9 of 10 adults face at least one form of ageism daily [3]. Initiatives like the WHO’s Global Campaign to Combat Ageism, which aims to mitigate ageist attitudes in societal structures, are among efforts to address the adverse effects of ageism [4,5]. The American Association of Retired Persons Disrupt Aging campaign calls on gerontologists to research the aging process and mitigate negative stereotypes that hinder older adults’ quality of life [6].

Digital ageism, also called artificial intelligence (AI) ageism, describes the manifestation of structural age-related bias in technologies like AI systems trained on uncurated data collected from public and private sources [7]. AI technologies are not inherently biased; they reflect and perpetuate society’s prejudicial attitudes and discriminatory practices toward older adults [8]. In the context of AI technologies, these structural and algorithmic biases can emerge in content that excludes or misrepresents older adults’ habits, interests, and values [9-11]. The ethical implications of these biases require AI developers and researchers to develop strategies that promote the critical evaluation of data collection to ensure equitable, inclusive, and patient-centered representation of populations, especially since AI tools influence public perceptions of aging and serve as tools for geriatric education and health care delivery [12].

Research interest in algorithmic bias in AI-generated content has recently increased, with emerging attention to how AI depicts age and reinforces ageist narratives [13,14]. The older adults portrayed in AI-generated imagery do not represent actual people; instead, they reproduce normative hierarchies, perpetuate stereotypes about aging, and generalize deficit-based perceptions of older adulthood [15,16]. Analyses of the representation of older adults in other media illustrate the effects of these normalized hierarchies and stereotypes. For example, the representation of older adults in human-robot interaction has been found to reinforce views of the aging body as broken and burdensome [15]. Consistent exposure to predominantly negative representations of aging in media may lead older adults to internalize negative self-imagery, which can detrimentally affect their health, self-efficacy, confidence, and even life expectancy [16,17].

In this study, we examined images generated by DALL-E 2, OpenAI’s text-to-image generator [18], at 2 points 1 year apart, using identical text prompts from the geriatric lexicon. We hypothesized that the AI image generator would produce fewer representations of visual characteristics resembling digital ageism over time in images depicting geriatric concepts, evidenced through increased diversity in demographic categories and increased positive emotions depicted in facial expressions.

## Methods

### Image Generation

In October and November 2022, 1 month after it became publicly available [19], we used the DALL-E 2 image generator to produce 164 images using 31 natural language text prompts from the geriatric lexicon. We selected DALL-E 2 because it was the leading generative AI text-to-image deep learning technology available to the American public without a paid subscription in 2022. Other image generators, such as Midjourney, were available through a paid subscription. The second batch of images was generated in September 2023, just before DALL-E 2 transitioned to an upgraded model (DALL-E 3). We generated images 1 year apart to ensure continuity by using DALL-E 2 at 2 points in time, following its lifespan. Although OpenAI has not released the source code for DALL-E 2, it used public datasets and created images by running a text prompt through OpenAI’s language models CLIP (contrastive language-image pretraining) and unCLIP and then through a separate diffusion model [20].

During data collection in 2022, DALL-E 2 offered users free tokens, which renewed each month, to create images. Each text prompt used a token, resulting in 4 images. We selected DALL-E 2 because of its accessibility as a free, publicly available tool, so we limited image generation to the token limit, which resulted in 164 images. Prompts were created by a geriatrician and included gendered terms (eg, *old man*), gender-neutral terms (eg, *elderly*), descriptive terms (eg, *lively old woman*), and geriatric conditions (eg, *dementia*) (Table S1 in Multimedia Appendix 1). The prompts were expression-neutral, with only 2 of the 31 prompts containing emotional descriptors. We standardized the prompts by restricting the text to a short phrase or single word without descriptive language or specification of an artistic style. Several prompts were entered 2 or 3 times to balance the prevalence of gender-specific and non–gender-specific prompts.

The same 31 prompts were used again in September 2023 to generate another 164 images. Examples of generated images are shown in Multimedia Appendix 2. After excluding images without recognizable facial expressions from both batches, our final sample included 153 images from 2022 and 147 images from 2023. Examples of excluded imagery and imagery without recognizable facial expressions are included in Multimedia Appendix 3.

### Expression Scoring

A team of 10 evaluators was selected purposively to include the authors of this article and medical students. The team comprised 2 faculty members with expertise in geriatrics, visual art, and AI literacy and 8 medical students studying geriatrics. Demographically, there were 80% White, 10% American Indian/Alaskan Native, and 10% Asian participants among the evaluators, with 80% being female. The evaluators participated in a norming session to establish consistent image analysis criteria and ensure interrater reliability. They analyzed images for gender (male or female), race (White; Asian; Black/African American; American Indian/Alaskan Native; Native Hawaiian; and unknown, including unclear or multiracialized; categories besides White and Asian were later combined as “other” due to low numbers), socioeconomic status (upper class, middle class, lower class, and unknown, subjectively determined based on the individual’s clothing and context as depicted in the image), primary emotion evident in facial expressions (positive [happy], neutral [calm, relaxed, and neutral], and negative [sad, nervous, angry, in pain, frustrated, worried, and confused]), and unknown (for unclear expressions, as in a blurred image).

During the norming session, the evaluators clarified that “Asian” encompassed representations of East Asian, South Asian, and Indian racialized figures, and the category “unknown” indicated representations with mixed racial identity or otherwise ambiguous classification. Disagreements were resolved during the session by reviewing examples and sharing reasoning for the visual assessments.

Using conceptual content analysis, the authors independently analyzed the images from both years. Based on their visual judgment, they recorded observations of perceived representations of gendered, racialized, and socioeconomic characteristics and primary emotional expressions by inputting a tally mark for each characteristic into a Microsoft Excel spreadsheet.

### Ethical Considerations

Since the subjects of this study were AI-generated images, ethics approval from the institutional review board was not sought.

### Statistical Analysis

Mean scores were based on counts of perceived representations of demographic and primary emotion categories for the paired samples. To test if the AI generator was adjusting ageism factors in images, comparisons between the years 2022 and 2023 were necessary for each factor: sex, race, socioeconomic status, and emotional expression. We chose a 2-way ANOVA to test these differences and included interaction (type III) to look for instances where there might be a yearly difference in representation in the images. The normality of the residuals was tested using the D’Agostino-Pearson omnibus (K2), Anderson-Darling (A2*), Shapiro-Wilk (W), and Kolmogorov-Smirnov (distance) tests. No nonnormality was found. Secondary effects were compared using independent 2-tailed *t* tests for groups of 2 or with Tukey multiple comparison adjustment for groups of 3 or more. An α of .05 was selected to indicate significance. Analyses were done using GraphPad Prism (version 10.0.0; GraphPad Software) for Windows.

### Data Exclusion

Generated images that did not include representations of people, images where emotional expression could not be identified, and images of younger people were excluded from the study. Examples of included images are shown inMultimedia Appendix 2 and excluded images are shown in Multimedia Appendix 3.

## Results

### Gender Representation

**Figure 1.**
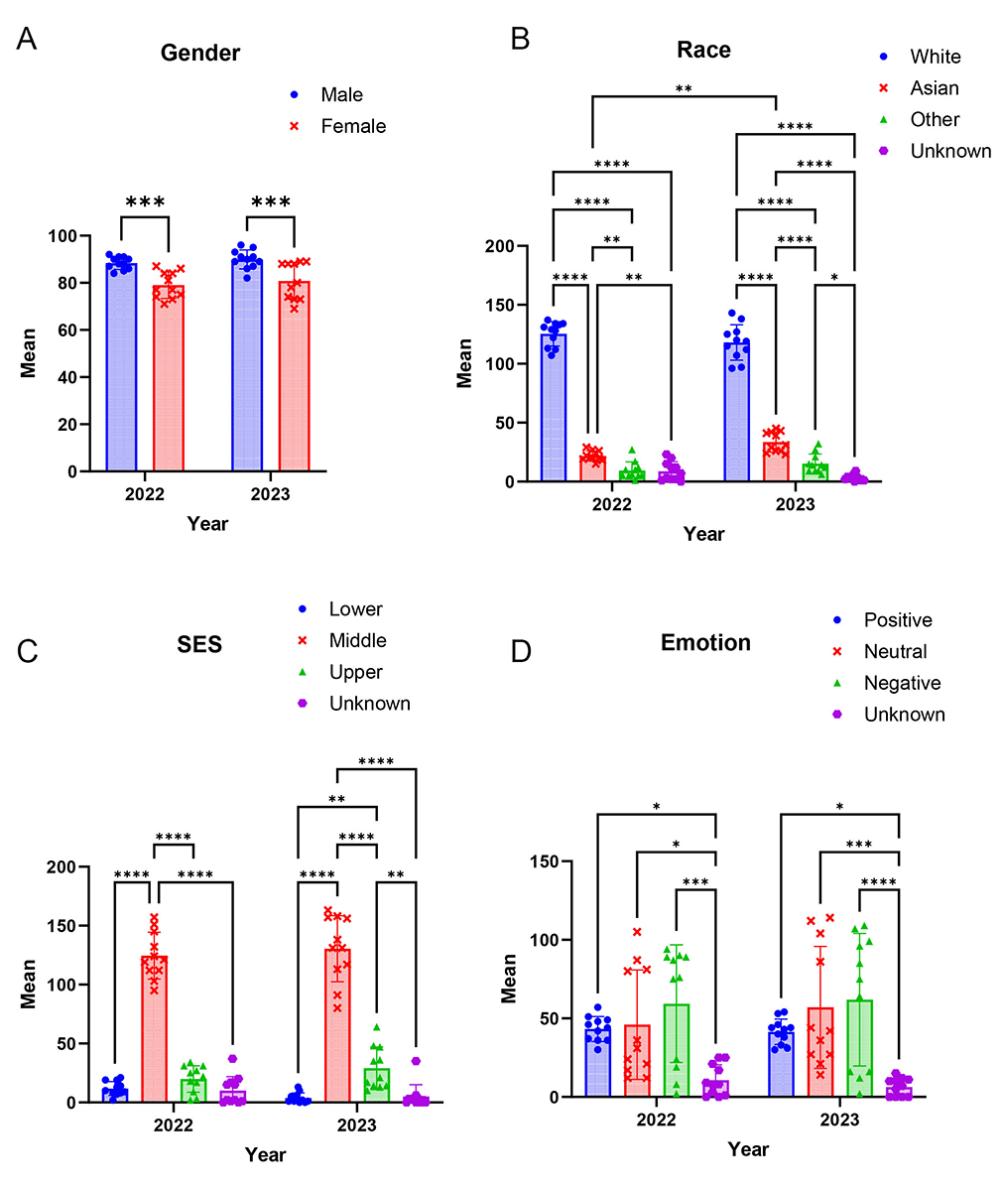
Analyses of images generated by artificial intelligence for sex, race, socioeconomic status, and emotion over 2 time points, 1 year apart. The graphs are representations of the means of scores from 10 independent analysts who scored the images for categories of (A) gender, (B) race, (C) socioeconomic status (SES), and (D) emotion. Data are represented as the mean (SD). *P<.05, **P<.01, ***P<.001, ****P<.0001.

When categorizing images by gender, we found no significant difference between years (*F*_1,40_=1.08; *P=*.31), though there was a mean difference in gender representation overall (*F*_1,40_=32.43; *P<*.001). As shown in Figure 1A, male representation was significantly higher than female representation in both 2022 (*t*_40_=4.09; *P<*.001) and 2023 (*t*_40_=3.97; *P*<.001). The prompts given for image generation had a male-to-female ratio of 12:5 (ie, 2.4:1), but the resulting images showed a ratio of approximately 1.2:1, indicating a significant decrease in the proportion of male images relative to the prompts (*z*=6.38; *P*<.001).

### Racialized Representation

Analysis of race categories (White, Asian, other, and unknown) indicated no significant difference between years in overall racial representation (*F*_1,80_=0.40; *P*=.53), but there was a difference in the average categorization of race (*F*_3,80_=834; *P<*.001). The interaction term was significant (*F*_3,80_=6.01; *P<*.001), suggesting variability in racialized representation by year. The Tukey test showed that all possible pairs of race categories were significantly different (*P<*.05) for both years, with White representation being 5-fold higher than other races in both years. There was a significant increase in Asian representation between 2022 and 2023 (*t*_80_=4.44; *P<*.01), as shown in Figure 1B.

### Socioeconomic Status

For socioeconomic status, (lower, middle, upper, and unknown), there was no significant difference between years (*F*_1,80_=0.02; *P=*.89), nor was the interaction term significant (*F*_3,80_=1.56; *P=*.20). There was a significant difference in representation by socioeconomic status (*F*_3,80_=304.1; *P<*.01). As shown in Figure 1C, middle-class representation was significantly higher than all other categories in both years (all *P<*.001). In 2023, upper-class representation was significantly higher than lower-class (*P*=.00) and other (*P*=.00) categories.

### Emotional Expression

Regarding emotional expression, there was no significant difference between years (*F*_1,80_=0.10; *P=*.75) or interaction with emotions (*F*_3,80_=0.32; *P=*.81). There were significant differences in mean emotion overall (*F*_3,80_=14.80; *P<*.001). As shown in Figure 1D, for both years, the “unknown” category was significantly less frequent than the negative (*P*<.001), neutral (*P=*.02 and *P*=.00), or positive (*P=*.04 and *P=*.02) categories. There were no significant differences between the frequencies of positive, neutral, and negative emotional expressions in either year.

## Discussion

### Principal Findings

This study investigated AI-generated images of older adults over time for evidence of potential changes in visual characteristics associated with digital ageism. Our hypothesis that the AI image generator would produce fewer representations of visual characteristics resembling digital ageism over time was not supported. The analysis of images generated using OpenAI’s Dall-E 2 in 2022 and 2023 revealed no significant difference in demographic or primary emotion categories between the 2 time points, except an increase in Asian-racialized figures in 2023. Instead, the images consistently reinforced demographic and emotional characteristic hierarchies associated with digital ageism. For gender representation, male representation remained higher than female representation, with no significant year-to-year variations. Regarding racialized representation, the images predominantly depicted White individuals in 2022 and 2023, with a statistically significant increase in the representation of Asian individuals and representations in the “other” category in 2023. For socioeconomic status, in both years, middle-class representation was significantly higher than other socioeconomic classes. Finally, for emotional expression, despite using predominantly neutral language in the prompts, the images represented negative primary emotions (angry, frustrated) over positive (happy) or neutral (relaxed) emotions, with no improvement toward more positive emotional representation over time.

The persistent generation of predominantly White-racialized, middle-class figures expressing negative emotions reinforces normative aging narratives, perpetuates negative portrayals of older adults’ well-being in digital contexts, and highlights the need for continued attention to ageism in AI-generated images. The hypothesis that text-to-image generators like DALL-E 2 are being trained successfully under large language models in order to decrease the prevalence of ageism and bias was not supported.

These findings demonstrate how ageism, originally defined by Butler [1] in 1969 and defined by the WHO as prejudice, stereotypes, and discrimination based on age, has persisted into digital media, manifesting as digital ageism in AI-generated content [1,2,7]. The findings confirm research that suggests generative AI technologies, although not inherently biased, reflect and perpetuate societal prejudiced attitudes that normalize aging narratives as an unfavorable process of decline, increased vulnerability, and limited diversity [8].

The findings also highlight ethical implications in developing and researching AI technologies [12]. Despite not representing real people, AI-generated images reproduce normative hierarchies, reinforce stereotypes about the aging body as broken and burdensome, and generalize deficit-based perceptions of older adulthood [15,16]. This suggests the need for developers, researchers, and users of AI-generated content to apply strategies that promote the critical evaluation of generated content to ensure equitable and inclusive representations of older adults [9-11]. These strategies are especially significant as AI-generated content influences public perceptions of aging and serves as a tool for geriatric education and health care delivery.

The persistence of visual characteristics of digital ageism in AI-generated images over the single year of our study suggests that the technology is not “learning” to produce more positive or diverse representations of aging. This is particularly concerning as consistent exposure to digital ageism through predominantly stereotyped representations may lead older adults to internalize negative self-imagery, which can detrimentally affect their health and life expectancy [16,17]. Conversely, positive images of aging can lead to improved health outcomes for older adults through increased confidence and self-efficacy [21,22].

Digital ageist narratives may also emerge in health care systems through paternalistic approaches and barriers to high-quality health care [17]. Although generative AI technologies can help produce health care plans for older adults, our findings suggest that practitioners should use these technologies critically to account for algorithmic biases that might perpetuate ageist narratives [23,24].

### Limitations

This study focused only on AI-generated imagery and did not examine other AI-generated content that may express digital ageism, such as written papers, advertisements, or music. We analyzed images generated only by OpenAI’s DALL-E 2 model, which is no longer available. Newer AI image generators may show different patterns of visual characteristics related to digital ageism. Additionally, one geriatrician identified text prompts that may reflect implicit bias. The 1-year data collection period may not have provided enough time to reflect changes in the AI datasets. Finally, image analysis relied on the authors’ visual judgements, which may reflect implicit biases. Having a more extensive and diverse group of evaluators might mitigate this potential source of bias.

### Comparison With Prior Work

A study examining the AI cycle of health inequity determined that digital ageism may contribute to systemic differences in health outcomes across different populations, and that contextual biases, not just technical biases, must be addressed in health care delivery [25]. A study on racial and sex diversity in ophthalmology using images generated by DALL-E 2 found that although the representations reflected increasing diversity among young professionals in the field, they also amplified existing biases [26]. The United Nations assessed AI-generated images of science, technology, engineering, and mathematics (STEM) professionals and found that 75% to 100% of generated images depicted men, which could negatively impact women’s self-perception in STEM fields [27]. Our study found that when generating images associated with aging, the gender gap, while still favoring men, was less pronounced than in the STEM study.

### Next Steps

This study found that the DALL-E 2 AI image generator perpetuated visual characteristics of digital ageism over time. Future research could replicate this study using newer AI text-to-image generation models and compare images generated across platforms. Including older adults as evaluators or forming interdisciplinary teams including gerontology experts in the image analysis process would provide valuable perspectives. Future research may also provide additional time between the generation of image batches, or several batches generated over a more extended period, to account for possible changes in AI datasets. Additionally, having multiple individuals from diverse backgrounds use the AI image generator to create images might reveal whether the output is influenced by the identity characteristics of the person initiating the generation. Finally, having a team of geriatricians identify more inclusive and representative text prompts may also mitigate implicit biases.

### Conclusions

Despite the increased social emphasis on positive views of aging, OpenAI’s DALL-E 2 image generator persistently generated images with visual characteristics of digital ageism by perpetuating limited diversity in representations of older adults and primarily depicting individuals with negative or neutral emotions. Given the expanding prevalence of AI technologies in health, education, and public health, it is essential to acknowledge and address the impact that digital ageism may have on older adults and geriatric care.

## Supplementary material

10.2196/68428 Multimedia Appendix 1 Text prompts entered in DALL-E for image generation.

10.2196/68428 Multimedia Appendix 2 Examples of generated images.

10.2196/68428 Multimedia Appendix 3 Examples of excluded imagery and imagery without recognizable facial expressions.

## Abbreviations

AI: artificial intelligence
CLIP: contrastive language-image pretraining
STEM: science, technology, engineering, and mathematics
WHO: World Health Organization
