# Supplementary material for: Recapitulation of Ageism in Artificial Intelligence–Generated Images: Longitudinal Comparative Study
Source: J Med Internet Res. 2025 Aug 13;27:e68428. doi: 10.2196/68428 (PMC12349884; doi:10.2196/68428)
Supplement: Multimedia Appendix 1 [file jmir-v27-e68428-s001.docx]

**Supplementary Table 1:** Text prompts entered in DALL-E for image generation.

| **Prompt** | **Entered X times** |
| --- | --- |
| Old Man | 2 |
| Memory Loss | 2 |
| Successful Aging | 3 |
| Lively Old Woman | 2 |
| Lively Old Man | 1 |
| Frail Older Adult | 2 |
| Frail Old Woman | 2 |
| Frail Old Man | 2 |
| Old Man Running | 1 |
| Old Man Weightlifting | 1 |
| Old Man Lifting Weights | 1 |
| Old Man and Car | 1 |
| Old Man and Cane | 2 |
| Old Man at Home | 1 |
| Old Woman at Home | 1 |
| Elderly | 1 |
| Geriatric | 1 |
| Aging 80 years | 1 |
| Frail Elderly | 1 |
| Functional Aging Person | 1 |
| Older Adult Intimacy | 1 |
| Life in Nursing Home Older Adult | 1 |
| Life in a Nursing Home | 1 |
| Driving Older Adult | 1 |
| Functional Impairment Old Age | 1 |
| Exceptional Lifespan | 1 |
| Blue Zone and Longevity Older Adult | 1 |
| Depression Older Adult | 1 |
| Delirium Older Adult | 1 |
| Dementia | 1 |
| Urinary Incontinence | 1 |
