# Supplementary figures and images for: Recapitulation of Ageism in Artificial Intelligence–Generated Images: Longitudinal Comparative Study
Source: J Med Internet Res. 2025 Aug 13;27:e68428. doi: 10.2196/68428 (PMC12349884; doi:10.2196/68428)

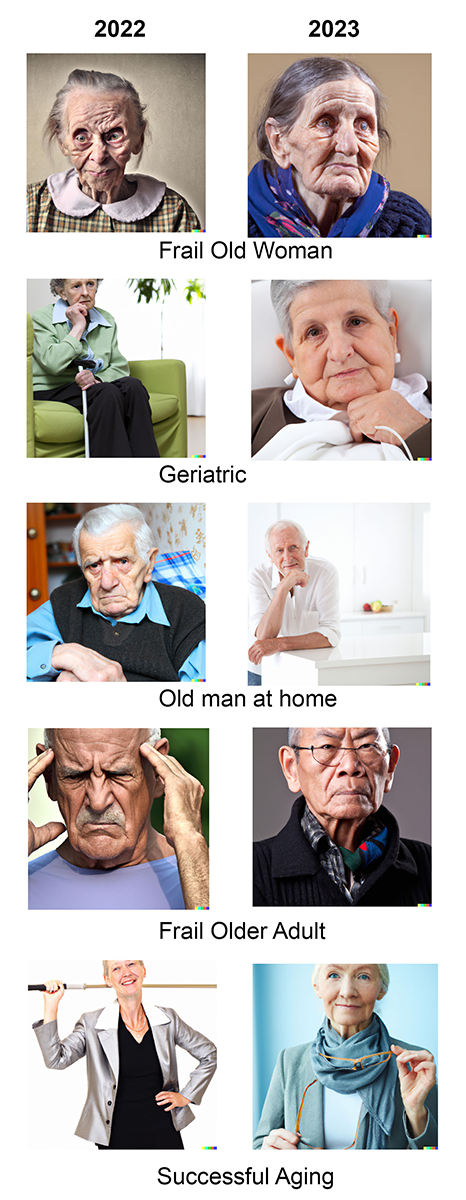

Supplement: Multimedia Appendix 2 [file jmir-v27-e68428-s002.png]

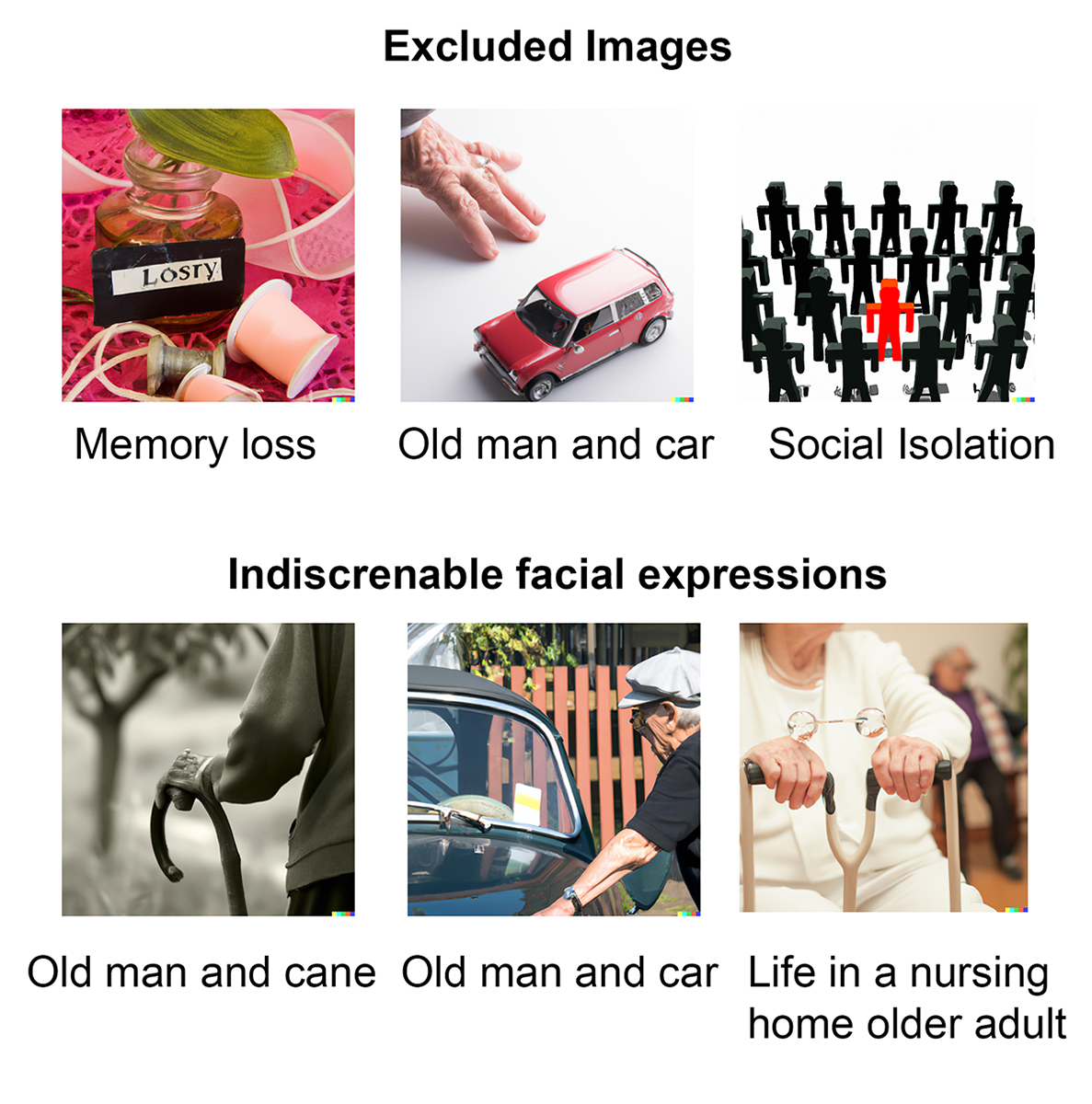

Supplement: Multimedia Appendix 3 [file jmir-v27-e68428-s003.png]
